# Supplementary material for: CD44 Receptor-Mediated/Reactive Oxygen Species-Sensitive Delivery of Nanophotosensitizers against Cervical Cancer Cells
Source: Int J Mol Sci. 2022 Mar 25;23(7):3594. doi: 10.3390/ijms23073594 (PMC8998256; doi:10.3390/ijms23073594)
Supplement: Supplementary file 1 [file ijms-23-03594-s001.zip › ijms-1591422-supplementary.pdf]

Supplementary materials

Experimental

Calibration curve of Ce6

To determine calibration curve of Ce6, Ce6 (10 mg) dissolved in 10 ml DMSO (1 mg/ml) was then this was diluted 100 times with DMSO/water mixed solvent (9/1, v/v). Absorbance of this solution was measured with Genesys 10s UV-VIS spectrophotometer (Thermo Fisher Scientific, Waltham, Massachusetts, USA). Calibration curve was measured at the range of 0.1 ~ 7 μg/ml of Ce6 concentration. For calibration curve in PBS, Ce6 (1mg/ml DMSO) was diluted with PBS more than 30 times. Calibration curve was evaluated at the range of 0.3 ~ 10 μg/ml of Ce6 concentration.

Confocal laser scanning microscope

3×10^5 HeLa cells were seeded in 6 well plates with cover glass. To this cell culture, free Ce6 or nanophotosenstitizers were treated for 2 h. To study the effect of CD44 receptor blocking, free HA (1.0 mg/ml) was pretreated to HeLa cells 30 min before nanophotosensitizer treatment. Cells were washed with PBS twice and then fixed with 4 % paraformaldehyde for 15 min at room temperature. These were washed with PBS twice and then immobilized with mounting solution (Immunomount, Thermo Electron Co., Pittsburgh, PA, USA). The cells were observed with confocal laser scanning microscope (LSM 800, Carl Zeiss Microscopy GmbH, Jena, Germany).

Results

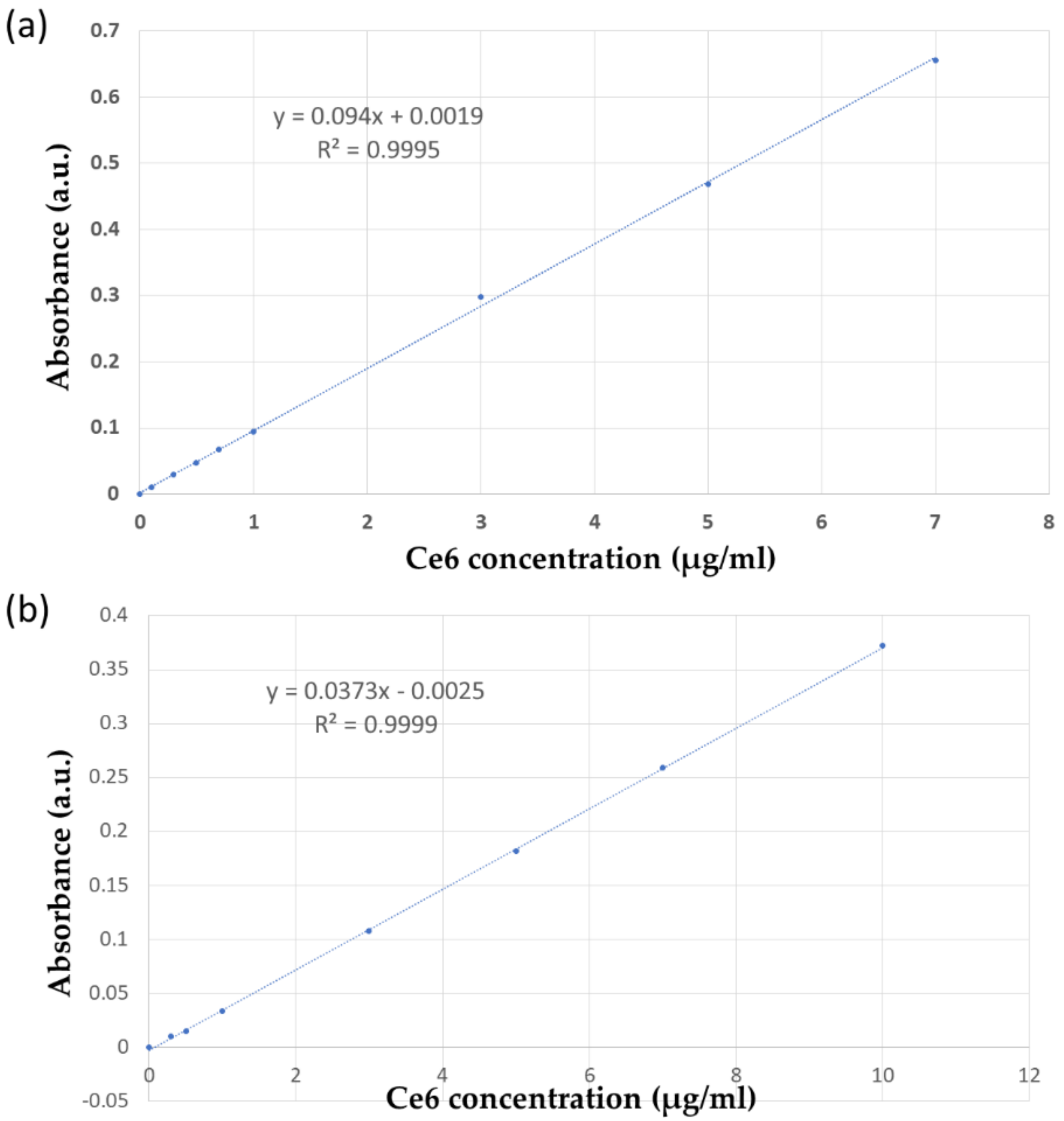

Figure S1. Calibration curve of Ce6. (a) DMSO/water, 9/1 (v/v); (b) Water. For calibration curve in DMSO, Ce6 (1mg/ml DMSO) was diluted with DMSO/water mixed solvent (9/1, v/v). For calibration curve, absorbance of this solution was measured with Genesys 10s UV-VIS spectrophotometer at 664 nm. Calibration curve was measured at the range of 0.1 ~ 7 μg/ml of Ce6 concentration. For calibration curve in PBS, Ce6 (1mg/ml DMSO) was diluted with PBS more than 30 times. Calibration curve was measured at the range of 0.3 ~ 10 μg/ml of Ce6 concentration.

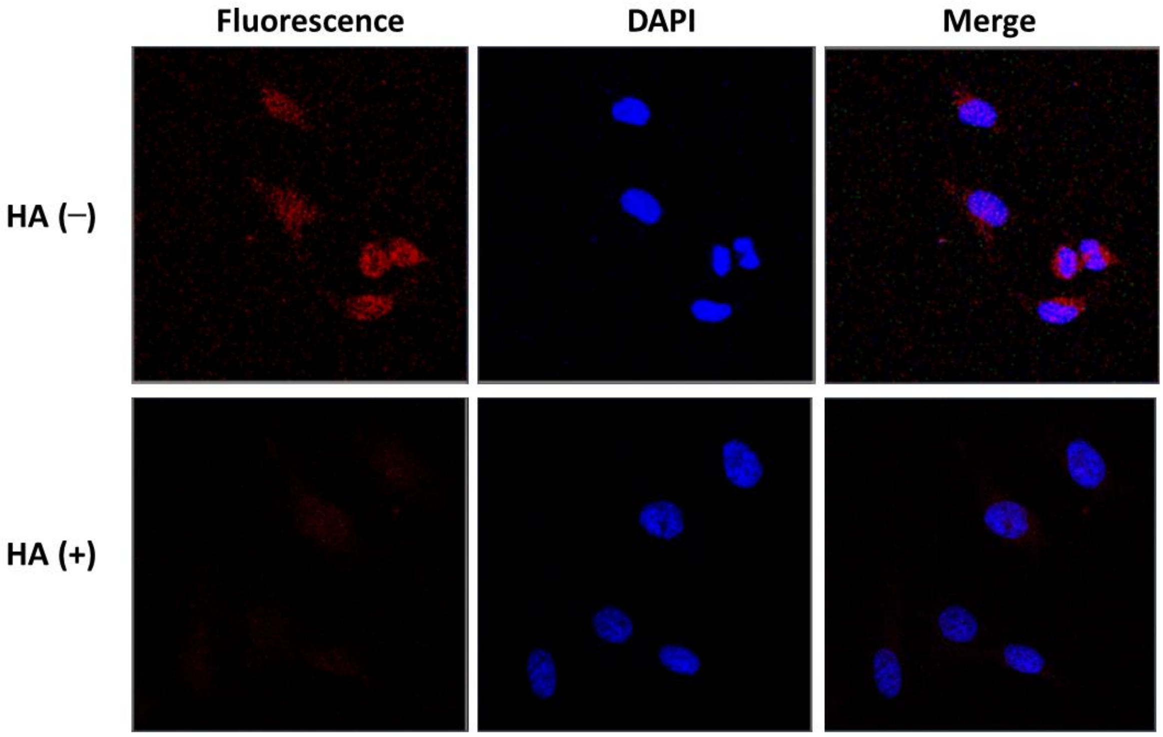

Figure S2. HeLa cells observed with confocal scanning microscope. The cells were pretreated with free HA as similar to Figure 5 and, after that, the nanophotosensitizers were treated. Magnification: 400 ×.
